# Gilteritinib-Associated Hypertriglyceridemia During Acute Myeloid Leukemia Triplet Therapy in a 52-Year-Old Man: A Case Report

**DOI:** 10.7759/cureus.111779

**Published:** 2026-06-29

**Authors:** Bhargav Vuppumalla, Steve Thomas, Karna Jagatheeswaran, Prabhath Surya Katta, Chaitanya Kumar Javvaji

**Affiliations:** 1 Medical Oncology, Sri Ramachandra Institute of Higher Education and Research, Chennai, IND; 2 Hematology, Sri Ramachandra Institute of Higher Education and Research, Chennai, IND; 3 Pediatrics, Motherness Fertility, Women and Children's Hospital, Bhimavaram, IND

**Keywords:** acute myeloid leukemia (aml), drug-interaction, gilteritinib, hypertriglyceridemia (tg), lipid metabolism

## Abstract

Acute myeloid leukemia (AML) harboring a FMS-like tyrosine kinase 3 internal tandem duplication (FLT3-ITD) mutation is characterized by a more aggressive clinical course, a higher likelihood of disease recurrence, and inferior survival outcomes. The introduction of targeted agents such as gilteritinib has expanded therapeutic options for patients with FLT3-mutated AML; however, the spectrum of rare treatment-related toxicities remains incompletely defined. We describe a case of profound hypertriglyceridemia that developed during treatment with a combination of azacitidine, venetoclax, and gilteritinib. A 52-year-old man presented with constitutional symptoms, marked leukocytosis, splenomegaly, and bone marrow findings consistent with AML exhibiting monocytic differentiation. Molecular analysis identified coexisting FLT3-ITD, nucleophosmin 1 (NPM1), and DNA methyltransferase 3A (DNMT3A) mutations. Owing to concomitant fungal pneumonia and unsuitability for intensive induction chemotherapy, he was treated with a modified regimen comprising azacitidine, venetoclax, and gilteritinib. The patient's baseline lipid profile was within normal limits. Following two cycles of induction therapy, a bone marrow aspirate was done. Serum triglyceride levels were found to be severely elevated at 6,964 mg/dL. Given the patient's prior episode of pancreatitis, this abnormality was considered clinically significant. Gilteritinib, venetoclax, and isavuconazole were discontinued, and lipid-lowering therapy with rosuvastatin and fenofibrate was commenced. Triglyceride concentrations progressively declined and returned to near-normal levels within one month. This report draws attention to a potentially serious and insufficiently recognized metabolic complication associated with FLT3 inhibitor-based combination therapy. It also underscores the importance of routine lipid surveillance, particularly in patients receiving concomitant azole antifungal agents.

## Introduction

Acute myeloid leukemia (AML) is a clonal malignancy arising from hematopoietic stem or progenitor cells. Genetic alterations affecting pluripotent hematopoietic stem cells or early myeloid precursors result in impaired cellular differentiation and uncontrolled proliferation of immature myeloid cells. The biological and molecular heterogeneity of AML has led to the development of increasingly personalized diagnostic and therapeutic approaches. Advances in understanding the molecular mechanisms underlying leukemogenesis have facilitated the emergence of targeted treatment strategies directed at specific genetic abnormalities [1,2].

The outcomes of AML treatment vary according to patient age and disease characteristics. Complete remission rates following initial therapy generally range between 60% and 80% in younger adults but decline to approximately 40%-60% among patients aged 65 years and older. Despite achieving remission, many patients eventually experience disease recurrence, which remains a major cause of treatment failure and mortality [3]. Progress in developing effective therapies for numerous AML subtypes has been hindered by the marked genetic and biological diversity of the disease. Among the molecular abnormalities associated with AML, mutations involving the FMS-like tyrosine kinase 3 (FLT3) gene are particularly important because they are consistently associated with adverse clinical outcomes and increased relapse risk [4].

FLT3 is a receptor tyrosine kinase that plays a critical role in normal hematopoiesis. The most common pathogenic alteration, the internal tandem duplication (ITD) mutation, occurs within the juxtamembrane region of the receptor. This mutation abolishes normal regulatory control, resulting in constitutive kinase activation independent of ligand binding. Continuous receptor signaling promotes leukemic cell proliferation, survival, and disease progression. Activated FLT3-ITD stimulates multiple downstream signaling cascades that contribute to leukemogenesis. Persistent activation of the Signal Transducer and Activator of Transcription 5 (STAT5) pathway enhances cellular proliferation and resistance to apoptosis. Concurrent activation of the phosphoinositide 3-kinase (PI3K)/protein kinase B (AKT)/mechanistic target of rapamycin (mTOR) signaling axis supports cell growth, survival, and metabolic adaptation. In addition, stimulation of the mitogen-activated protein kinase (MAPK)/extracellular signal-regulated kinase (ERK) pathway further promotes the expansion of malignant myeloid cells [3].

The presence of an FLT3-ITD mutation is widely recognized as a marker of poor prognosis in AML. Patients harboring this mutation exhibit a greater likelihood of relapse and reduced survival compared with those lacking FLT3 alterations [5]. Consequently, therapeutic efforts have focused on inhibiting aberrant FLT3 signaling. Tyrosine kinase inhibitors (TKIs) represent a class of targeted agents designed to block intracellular kinase activity, thereby interrupting oncogenic signaling pathways and suppressing tumor growth. Beyond intrinsic genetic abnormalities, AML progression is influenced by interactions between leukemic cells and the bone marrow microenvironment. Leukemic stem cells (LSCs), characterized by their capacity for self-renewal, impaired differentiation, and leukemia-initiating potential, are considered central drivers of disease persistence and recurrence [6]. In FLT3-ITD-positive AML, these therapy-resistant LSC populations are believed to contribute significantly to treatment resistance and post-remission relapse.

For many years, standard AML therapy consisted of remission induction with an anthracycline combined with cytarabine, followed by consolidation using high-dose cytarabine and, when appropriate, allogeneic hematopoietic stem cell transplantation. Recognition of the pivotal role of FLT3 mutations in AML pathogenesis prompted the development of targeted inhibitors to complement or replace conventional chemotherapy approaches. Gilteritinib, a second-generation tyrosine kinase inhibitor, exhibits activity against several kinases, including FLT3, AXL receptor tyrosine kinase (AXL), Anaplastic Lymphoma Kinase (ALK), and c-KIT. Importantly, gilteritinib demonstrates potent inhibition of both FLT3 internal tandem duplication (ITD) and tyrosine kinase domain (TKD) mutations, including FLT3-ITD, FLT3-ITD-D835Y, and FLT3-D835Y variants. Through suppression of constitutively activated FLT3 signaling, gilteritinib promotes apoptosis and inhibits the proliferation of FLT3-mutated leukemic cells, thereby improving therapeutic outcomes in this high-risk AML subgroup.

## Case presentation

A 52-year-old man presented to Sri Ramachandra Institute of Higher Education and Research with complaints of progressive fatigue, recurrent episodes of fever, and night sweats. His medical history was notable for an episode of acute pancreatitis complicated by a peripancreatic collection in 2018, which had been managed successfully. Clinical examination revealed pallor and significant splenomegaly, measuring 21 cm on ultrasonography. No other remarkable findings were identified on physical examination.

Initial laboratory investigations demonstrated marked leukocytosis. Peripheral blood smear examination showed normocytic normochromic erythrocytes with a pronounced left shift and approximately 90% circulating blasts, raising suspicion for acute leukemia. Bone marrow aspiration revealed a markedly hypercellular marrow with blasts accounting for nearly 95% of nucleated cells. Immunophenotypic characterization by flow cytometry and immunohistochemistry identified a cluster of differentiation (CD) 45-dim, low side-scatter blast population expressing CD13, CD33, CD36, CD11c, CD117, CD38, CD58, Human Leukocyte Antigen - DR isotype (HLA-DR), and myeloperoxidase (MPO), while lacking lymphoid lineage markers. These findings were diagnostic of AML. Bone marrow biopsy further demonstrated diffuse replacement of the marrow by sheets of atypical blast cells within a hypercellular background, confirming the diagnosis.

**Figure 1:**
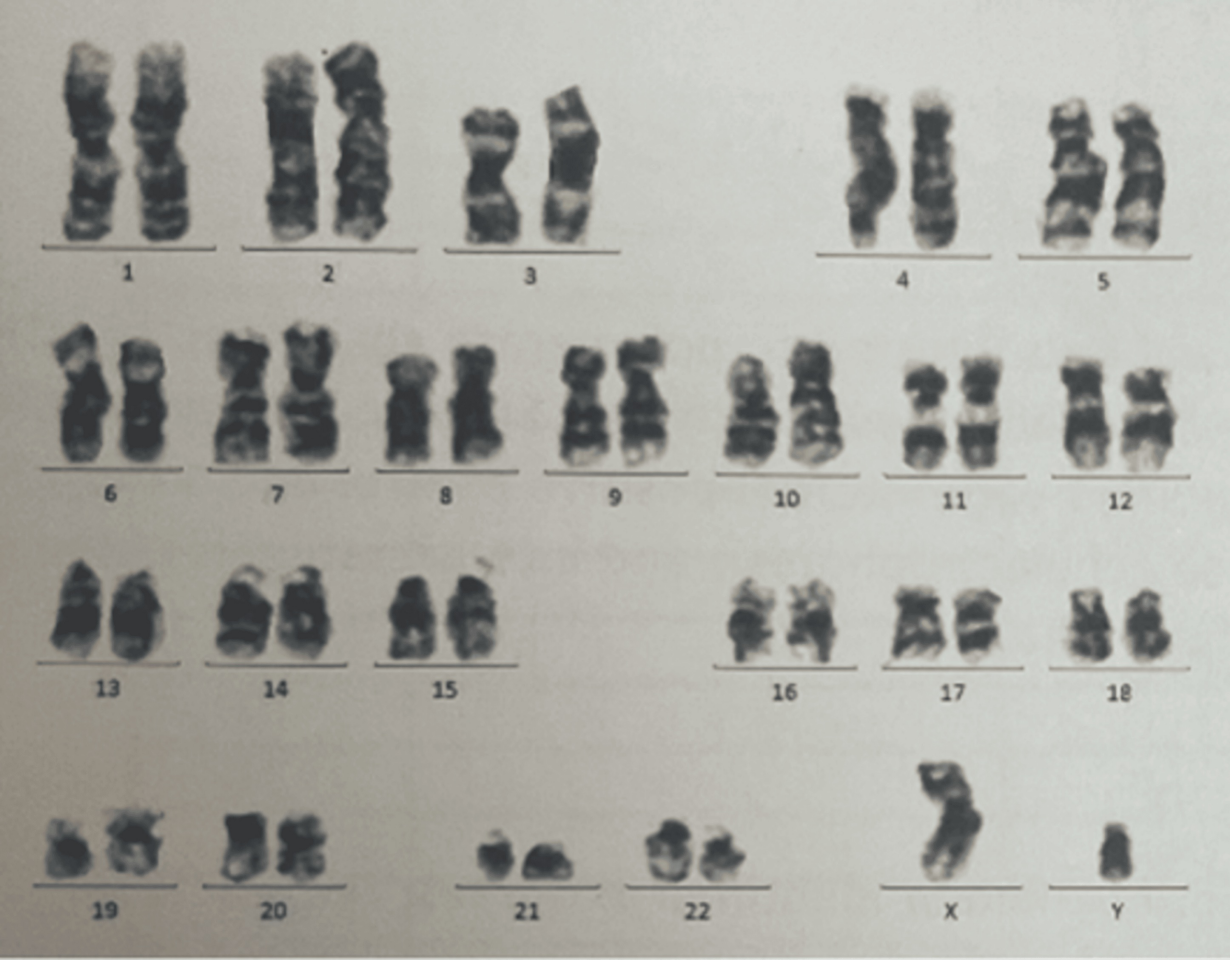
Chromosomal structural variations by karyotyping showing normal male karyotype in 25 metaphases in a neoplastic sample

Conventional cytogenetic analysis revealed a normal male karyotype across 25 analyzed metaphases (Figure 1). Molecular profiling using next-generation sequencing (NGS) identified FLT3-ITD p.Gly583_Leu601dup (in-frame insertion; variant allele frequency {VAF} (31.88%), DNMT3A p.Arg882His (VAF 46.6%), and NPM1 p.Gln289LeufsTer11 (VAF 38.82%). Based on the morphologic, immunophenotypic, cytogenetic, and molecular findings, a diagnosis of AML with FLT3-ITD, NPM1, and DNMT3A mutations was established and categorized as intermediate-risk disease.

Baseline biochemical investigations, including a fasting lipid profile, were within normal limits. Cardiac assessment with electrocardiography and transthoracic echocardiography showed preserved cardiac function with a left ventricular ejection fraction of 64%. Given the presence of hyperleukocytosis, cytoreduction with hydroxyurea was initiated. The patient's molecular and cytogenetic findings were subsequently reviewed at a multidisciplinary tumor board meeting to formulate an individualized treatment strategy.

At presentation, the patient was also found to have invasive fungal pneumonia, evidenced by a serum galactomannan index of 8.23 and the need for supplemental oxygen therapy. Intravenous voriconazole was commenced, resulting in gradual clinical stabilization. Owing to active fungal infection and poor suitability for intensive induction chemotherapy with the conventional “7+3” regimen, the patient was considered unsuitable for intensive treatment. Consequently, a combination regimen consisting of azacitidine, venetoclax, and gilteritinib (AZA-VEN-GILT) was selected.

The standard protocol included azacitidine 75 mg/m² administered subcutaneously or intravenously on days 1-7, venetoclax administered orally with dose escalation from 100 mg on day 1 to 200 mg on day 2 and 400 mg from day 3 onward, and gilteritinib 120 mg orally once daily on days 1-28 of each 28-day cycle. Following discussion by the institutional hemato-oncology board and consideration of the patient's age, body surface area, performance status, active infection, and financial limitations, a modified regimen was adopted. The patient received azacitidine 100 mg intravenously on days 1-7, venetoclax 100 mg orally for eight days while receiving concurrent antifungal therapy, and gilteritinib 80 mg orally for 21 days in each 28-day cycle. Two induction cycles were completed using this modified schedule.

**Figure 2:**
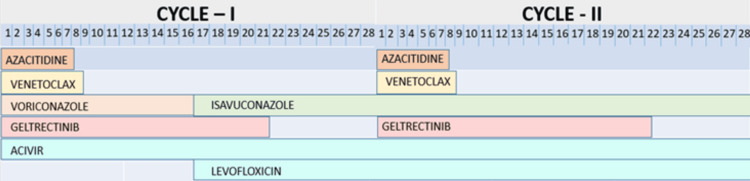
Treatment timeline of administration of azacitidine-venetoclax-geltrectinib regimen and supportive antimicrobial therapy across two treatment cycles

Comprehensive supportive care was maintained throughout treatment. Antimicrobial prophylaxis consisted of acyclovir, voriconazole, and levofloxacin according to institutional practice guidelines. The overall treatment course and antimicrobial administration are summarized in Figure 2. Following completion of the first treatment cycle, the patient developed a persistent cough. Bronchoscopy with bronchoalveolar lavage was performed, but no significant abnormalities were identified. Given the previous diagnosis of fungal pneumonia, antifungal therapy was transitioned to oral isavuconazole 200 mg daily, after which respiratory symptoms improved substantially.

At the conclusion of the second treatment cycle, bone marrow aspiration obtained from the posterior superior iliac spine demonstrated an unusual appearance. The aspirate specimen exhibited distinct two-layer separation, with a prominent milky-white supernatant overlying the marrow sample. This unexpected finding prompted evaluation for an underlying lipid abnormality. Repeat biochemical testing revealed profound hypertriglyceridemia, with serum triglyceride levels measuring 6,964 mg/dL. Lipid-lowering treatment with rosuvastatin and fenofibrate was initiated immediately.

Because AML is not commonly associated with severe hypertriglyceridemia, a treatment-related adverse effect was considered the most likely explanation. After causality assessment, gilteritinib, venetoclax, and isavuconazole were identified as the most probable contributors and were discontinued. Although severe hypertriglyceridemia has not been definitively linked to any of these agents individually, a pharmacologic interaction among the combination regimen may have contributed to the development of this metabolic complication.

Serial lipid profiles were obtained on a weekly basis to monitor response. As shown in Table 1, triglyceride concentrations progressively declined and returned toward normal levels over the subsequent month. Following discussion of treatment options and consideration of the patient's preferences, gilteritinib was permanently withdrawn from the treatment regimen. No subsequent episodes of hypertriglyceridemia were documented during follow-up.

**Table 1:** Weekly monitoring of lipid profile showing gradual normalization of triglyceride levels over one month

Week	Triglycerides	Total cholesterol
1	6964	776
2	1357	421
3	317	180
4	216	126
5	184	98

## Discussion

Gilteritinib is generally well tolerated; however, several uncommon but clinically significant adverse effects have been reported, including febrile neutropenia, severe infections, pancreatitis, QT interval prolongation, and posterior reversible encephalopathy syndrome (PRES) [7]. To the best of our knowledge, no published reports have described severe hypertriglyceridemia associated with the azacitidine-venetoclax-gilteritinib combination regimen. The absence of prior reports may be attributable to the relatively recent incorporation of this triplet regimen into the therapeutic landscape of FLT3-mutated AML.

Isavuconazole is a broad-spectrum triazole antifungal frequently used in immunocompromised patients, including those undergoing treatment for hematologic malignancies. Although the exact mechanism by which isavuconazole influences lipid metabolism remains incompletely understood, alterations in hepatic metabolic pathways have been proposed. Isavuconazole acts as a moderate inhibitor of cytochrome P450 3A4 (CYP3A4), an enzyme involved in the metabolism of numerous endogenous and exogenous compounds. Through its interaction with hepatic enzyme systems, the drug may interfere with lipid homeostasis and contribute to elevations in serum triglyceride levels [8,9].

A direct causal association between the combined use of gilteritinib and isavuconazole and the development of severe hypertriglyceridemia has not been established. Nevertheless, a clinically relevant pharmacokinetic interaction may exist. Gilteritinib is predominantly metabolized via the CYP3A4 pathway, and exposure to the drug is known to increase when administered concurrently with CYP3A4 inhibitors. Although this interaction has been most clearly demonstrated with potent inhibitors such as itraconazole, the moderate CYP3A4 inhibitory activity of isavuconazole may similarly increase systemic gilteritinib concentrations, potentially enhancing toxicity. Whether elevated gilteritinib exposure contributes directly to disturbances in lipid metabolism remains uncertain and warrants further investigation.

Venetoclax has also been associated with metabolic abnormalities, including hypertriglyceridemia, in clinical studies involving patients with acute leukemias, particularly when administered as part of intensive combination regimens [10]. While hypertriglyceridemia has been described in certain hematologic malignancies, especially acute promyelocytic leukemia, it is not a recognized manifestation of AML itself, particularly in patients demonstrating a favorable therapeutic response [11]. Therefore, the marked elevation in triglyceride levels observed in our patient was considered unlikely to be attributable solely to the underlying leukemia.

A limitation of this report is the inability to definitively identify the individual agent responsible for the adverse drug reaction. Because gilteritinib, venetoclax, and isavuconazole were administered concurrently, the relative contribution of each drug remains unclear. Furthermore, a pharmacokinetic or pharmacodynamic interaction among these agents cannot be conclusively demonstrated based on a single case observation [12]. Nevertheless, the temporal relationship between therapy administration, the onset of severe hypertriglyceridemia, and the subsequent normalization of triglyceride levels following drug withdrawal strongly suggests a treatment-related etiology.

Management of the adverse event was successful with prompt initiation of lipid-lowering therapy using rosuvastatin and fenofibrate, resulting in progressive normalization of serum triglyceride concentrations. During subsequent treatment planning, the potential for clinically significant drug interactions was carefully considered. Posaconazole, a potent CYP3A4 inhibitor commonly used for antifungal prophylaxis in AML, was deliberately avoided because of concerns regarding increased gilteritinib exposure and the possibility of exacerbating treatment-related toxicity.

Based on clinical assessment and causality evaluation using the Naranjo Adverse Drug Reaction Probability Scale [13], the reaction was categorized as a possible adverse drug reaction. Although definitive evidence implicating a single agent was lacking, our multidisciplinary hemato-oncology team considered gilteritinib, particularly when administered in combination with venetoclax and/or isavuconazole, as the most plausible contributor. This case highlights the need for increased awareness of potentially serious metabolic complications associated with FLT3 inhibitor-based combination therapy. Regular monitoring of lipid parameters may be advisable in patients receiving gilteritinib alongside azole antifungals or other agents with potential metabolic interactions.

## Conclusions

This case highlights a rare and potentially serious occurrence of severe hypertriglyceridemia in a patient with acute myeloid leukemia receiving azacitidine-venetoclax-gilteritinib triplet therapy along with Isavuconazole. Although the exact etiological mechanism could not be definitively established, the temporal relationship between drug administration and onset of hypertriglyceridemia, followed by resolution after discontinuation of the suspected agents, suggests a possible treatment-related adverse drug reaction. A pharmacokinetic interaction mediated through CYP3A4 inhibition may have contributed to increased gilteritinib exposure and subsequent metabolic dysregulation. This case adds important clinical insight into the evolving safety profile of FLT3 inhibitor-based combination therapies in AML. Early recognition of this complication is clinically relevant, particularly in patients with prior pancreatic disease, as untreated severe hypertriglyceridemia may predispose to acute pancreatitis and other life-threatening complications. Regular lipid profile monitoring should be considered in patients receiving gilteritinib-based combination regimens, especially when co-administered with azole antifungals. Further pharmacovigilance studies and additional case reports are required to better characterize the incidence, mechanism, and risk factors associated with this uncommon adverse event.
